# Supplementary material for: Endothelial Function and Hypoxic–Hyperoxic Preconditioning in Coronary Surgery with a Cardiopulmonary Bypass: Randomized Clinical Trial
Source: Biomedicines. 2023 Mar 28;11(4):1044. doi: 10.3390/biomedicines11041044 (PMC10136033; doi:10.3390/biomedicines11041044)
Supplement: Supplementary file 1 [file biomedicines-11-01044-s001.zip › biomedicines-2249887-supplementary.pdf]

biomedicines

Supplementary Materials

Article

Endothelial function and Hypoxic-hyperoxic preconditioning in coronary surgery with a cardiopulmonary bypass: Randomized Clinical Trial

Irina A. Mandel [1,8,*], Yuriy K. Podoksenov [2,3], Sergey L. Mikheev [4,5], Irina V. Suhodolo [6], Yulia S. Svirko [7], Vladimir M. Shipulin [2,3], Anastasia V. Ivanova [9], Andrey G. Yavorovskiy [1] and Andrey I. Yaroshetskiy [10]

* **Correspondence:** Irina A. Mandel, e-mail: mandel_i_a@staff.sechenov.ru;

S1. The intraoperative anaerobic threshold measurement technique

The determination of the anaerobic threshold at the beginning of the operation in the background of anesthesia, mechanical ventilation, and myoplegia was carried out according to a specially developed method (RF Patent for Invention No. 2432114) [Method for Evaluating Degree of Metabolic and Cardiorespiratory Adaptation of Patient. RU 2432114 C1; 27.10.2011 Bull. 30; https://worldwide.espacenet.com/publicationDetails/biblio?CC=RU&NR=2432114C1&KC=C1&FT=D]. The data obtained were compared with the classical method of measuring the anaerobic threshold during ergospirometry carried out 72 hours before the operation.

The developed method did not require the use of complex expensive equipment (ergospirometer, device for preparing a hypoxic gas mixture) and allowed us to determine the anaerobic threshold during surgery.

A method was used for assessing the degree of metabolic and cardiorespiratory adaptation of cardio surgical patients by the power of the anaerobic threshold, including sequential inhalation of a gas mixture with 51% ($FiO_2 = 0.51$) and 21% ($FiO_2 = 0.21$) oxygen content. At the same time, it is necessary that the parameters of mechanical ventilation, indicators of homeostasis, and therapeutic measures remained unchanged throughout all measurements. At the inhalation of a gas mixture with a 51% oxygen content, the percentage of oxygen and the partial tension of carbon dioxide in the exhaled air were measured using the gas module of the Primus (Dräger) anesthesia respiratory apparatus. After the stabilization of indicators (usually after 3–5 minutes), their values were recorded, and oxygen consumption was determined according to Formula 1:

$$VO_2\text{–}0.51 = (51 - O_2 \text{ exp.- } 0.51) \times MV, \quad (1)$$

where

VO_2–0.51 - (ml/min) oxygen consumption by inhaling a gas mixture with $FiO_2 = 0.51$;

51 – (%) percentage of inspiratory oxygen when inhaling a gas mixture with FiO_2= 0.51;

O_2 exp. - 0.51 - (%) percentage of expiratory oxygen when inhaling a gas mixture with $FiO_2 = 0.51$;

MV – (ml/min) minute ventilation.

The release of carbon dioxide was determined by Formula 2:

$$VCO_2\text{-}0.51 = etCO_2\text{-}0.51 \times MV : BP, \quad (2)$$

where

VCO_2-0.51 - (ml/min) release of CO2 upon inhalation of a gas mixture with FiO2 = 0.51;

$etCO_2$-0.51 (mm Hg) partial pressure of CO_2 at the end of exhalation (end tidal) when inhaling a gas mixture with FiO_2 = 0.51;

MV – (ml/min) minute ventilation;

BP - (mm Hg) - Atmospheric pressure (barometric pressure).

After the measurements were completed, we proceeded with the inhalation of a gas mixture with a 21% oxygen content, and the percentage of oxygen and the partial pressure of carbon dioxide in the exhaled air were measured. According to the formulas below, after stabilization (usually after 3–5 minutes), oxygen consumption and carbon dioxide emission were determined after stabilization.

Formula 3:

$$VO_2\text{–}0.21 = (21 - O_2\ \text{exp.-}0.21) \times MV, \quad (3)$$

where

VO_2–0.21 - (ml/min) oxygen consumption by inhaling a gas mixture with FiO2 = 0.21;

21 – (%) п percentage of inspiratory oxygen when inhaling a gas mixture with FiO2 = 0.21;

O_2 exp. - 0.21 - (%) percentage of expiratory oxygen when inhaling a gas mixture with FiO_2 = 0.21;

MV – (ml/min) minute ventilation.

Formula 4:

$$VCO_2\text{-}0.21 = etCO_2\text{-}0.21 \times MV / BP, \quad (4)$$

where

VCO_2 - (ml/min) release of CO_2 upon inhalation of a gas mixture with FiO_2 = 0.21;

$etCO_2$-0.21 (mm Hg) partial pressure of CO_2 at the end of exhalation (end tidal) when inhaling a gas mixture with FiO_2 = 0.21;

MV – (ml/min) minute ventilation;

BP - (mm Hg) - Atmospheric pressure (barometric pressure).

Since the anaerobic threshold was determined at the moment of the intersection of the curves of oxygen consumption and carbon dioxide emission, i.e., when the value of oxygen consumption corresponding to the

moment of reaching the anaerobic threshold (VO_2–AT) was equal to VCO_2 and if the carbon dioxide emission did not change during the measurement stages (which almost always takes place), the determination of the anaerobic threshold power was carried out according to Formula 5:

$$PAT = 0.21 – (VO_2–0.21 – VCO_2) \times 0.3 : (VO_2–0.51 - VO_2–0.21), \quad (5)$$

where
PAT – (FiO_2) power of anaerobic threshold;
0.21 – FiO_2 in the room air;
VO_2–0.21 – (ml/min) oxygen consumption by inhaling a gas mixture with FiO_2 = 0.21;
VCO_2 - (ml/min) release of CO_2;
0.3 - Δ FiO_2 = 0.51 – 0.21;
VO_2–0.51 - (ml/min) oxygen consumption by inhaling a gas mixture with FiO_2 = 0.51.

The calculation principle is shown in Figure 1. The values of VO_2 and VCO_2 found during measurements 1, 2 (at FiO_2 = 0.51) and 3, 4 (at FiO_2 = 0.21) were plotted on a graph, then the points VO_2–0.51 and VO_2-0.21 were connected with a straight line, and the line continued until it intersected with the VCO_2 line, which was built in the same way.

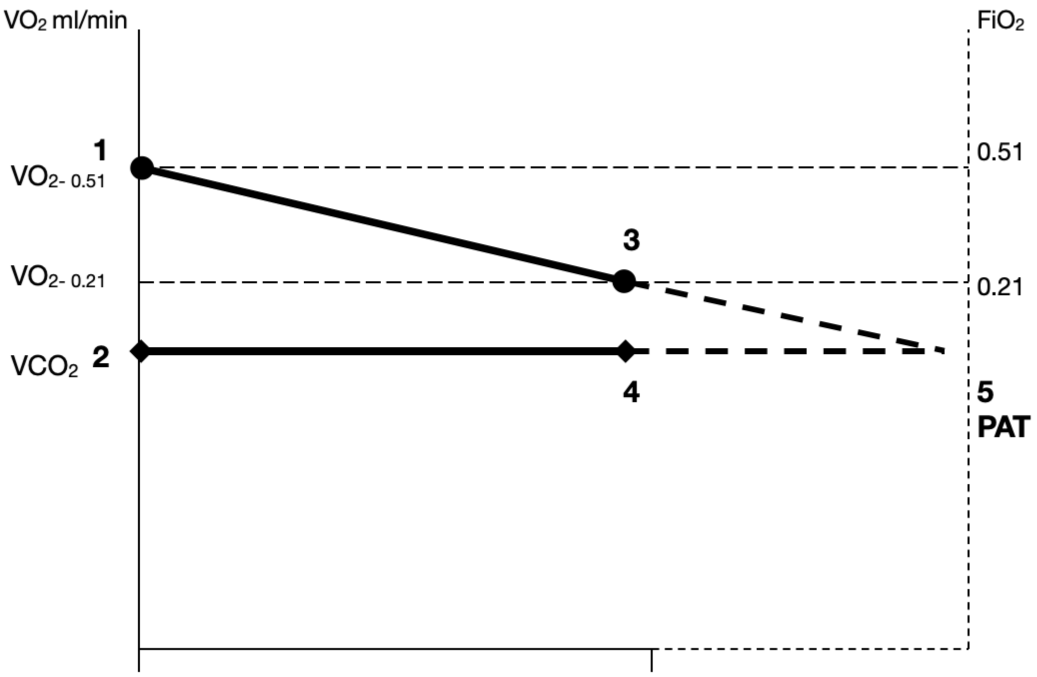

Supplementary Figure S1. Scheme for calculating the power of the anaerobic threshold. Numbers indicate the formulas’ numbers from the main text. FiO_2—oxygen fraction in the inhaled gas mixture, PAT—the power of the anaerobic threshold, VO_2—oxygen consumption, VCO_2—carbon dioxide release.

The values of VO_2 and VCO_2 found during measurements 1, 2 (at FiO_2 = 0.51) and 3, 4 (at FiO_2 = 0.21) were plotted on a graph, then the points VO_2–0.51 and VO_2-0.21 were connected with a straight line, and the line continued until it intersected with the VCO_2 line, which was built in the same way.

1. Supplementary Data

Supplementary Table S1. Dynamics of oxygen balance characteristics during hypoxic–hyperoxic preconditioning.

Parameter	Baseline	Hypoxic phase, *n*=60	Hyperoxic phase, *n*=60		
		10 min	10 min	20 min	30 min
CI, l/min/m^2	2.7 (2.4 -2.9)	2.75 (2.1 - 2.95)	2.75 (2.1 - 2.9)	2.75 (2.1 - 2.9)	2.75 (2.1 - 2.9)
paO_2, mm Hg	108.2 (104 - 120)	47.7 (46.5 - 49)*	342.0 (299.3 - 366)*	329.4 (290 - 379.6)*	330 (305.5 - 377.6)*
pvO_2, mm Hg	46.2 (44.8 - 47.4)	35.6 (32.7 - 38.8)*	62.5 (56.1 - 70)*	63.7 (57 - 69.1)*	62.5 (57 - 67)*
$paCO_2$, mm Hg	38.7 (34.73 - 42.18)	39 (36 - 43.2)	41.2 (37.2 - 47)	41.4 (38.5 - 43)	40 (38.9 - 45)
$pvCO_2$, mm Hg	44 (38.8 - 48)	43.0 (41.5 - 46.5)	45 (40.7 - 49.9)	44 (42.2 - 47.5)	47.6 (43 - 52)
ΔPCO_2, mm Hg	6 (2.9 - 6.9)	3.7 (2.7 - 6.75)*	3.0 (1.3 - 5.1)*	4.0 (2.8 - 5.5)*	5.7 (5.0 - 7.0)
CaO_2, ml/L	170.5 (161 - 185.2)	146 (128.3 - 162)*	173.1 (162 - 185.7)	173.4 (162 - 185.7)	173.4 (162 - 185.7)
CvO_2, ml/L	134.8 (122 - 144.9)	106.6 (93.3 - 115.5)*	148.2 (138.9 - 160.1)*	151.1 (139.8 - 164.6)*	147.9 (138.3 - 160.5)*
$C(a-v)O_2$, ml/L	35.46 (29.68 - 43.38)	40.15 (32.56 - 48.3)*	23 (15.9 - 26.02)*	20.11 (14.78 - 29.7)*	20.81 (17.72 - 26.36)*
ΔPCO_2/ $C(a-v)O_2$	1.56 (0.89 - 1.89)	0.97 (0.67 - 1.70)*	1.43 (0.73 - 2.38)	2.20 (0.92 - 3.12)*	2.49 (2.04 - 3.53)*
SaO_2, %	98.8 (97.6 - 99.4)	85 (82.6 - 86.6)*	99.9 (99.7 - 99.9)	99.9 (99.8 - 99.9)	99.9 (99.4 - 99.9)

SvO_2, %	77.4 (73.6 - 80.7)	59.1 (57 - 64.2)*	86 (84.4 - 88.7)*	87.4 (81.7 - 90.6)*	88 (83.5 - 88.9)*
IVO_2, ml/min/m^2	88.43 (72.09 - 110)	106 (87.8 - 117.2)*	59.7 (38.55 - 74.14)*	55.3 (39.8 - 71)*	55.5 (38 - 72.1)*
IDO_2, ml/min/m^2	440.7 (398.4 - 507.1)	367.8 (324.1 - 451.2)*	464.6 (390.8 - 532)	463.2 (390.8 - 532.4)	464.4 (390.8 - 532)
IEO_2, %	21.4 (17.24 - 25.33)	27.6 (25.02 - 32.6)*	13.9 (10.45 - 15)*	12.2 (8.21 - 18.22)*	11.9 (10.26 - 15.74)*
Lac, mM/l	1.45 (1.3 - 1.95)	1.8 (1.43 - 2.3)	1.65 (1.0 - 2.0)	1.7 (1.3 -2.1)	1.9 (1.5 - 2.3)
Glu, mM/l	6.1 (5.0 - 7.2)	5.8 (5.0 - 6.5)	6 (5.1 - 6.4)	5.7 (5.3 - 7.0)	6 (5.5 - 6.8)
rSO_2 left	62 (57 - 64)	51 (44 - 57)	72 (66 - 75)		
rSO_2 right	62 (56 - 65)	53 (42 - 58)	72 (67 - 78)		

CI—cardiac index; paO_2—arterial oxygen tension; pvO_2—mixed venous oxygen tension; $paCO_2$—arterial carbon dioxide tension; $pvCO_2$—venous carbon dioxide tension; ΔPCO_2—venous-to-arterial carbon dioxide difference; CaO_2—arterial oxygen content; CvO_2—venous oxygen content; $Ca\text{-}vO_2$—arterial–venous oxygen content; $\Delta PCO_2/Ca\text{-}vO_2$—venous-to-arterial carbon dioxide difference/arterial–venous oxygen content difference ratio; SaO_2—arterial oxygen saturation; SvO_2 — mixed venous oxygen saturation; IEO_2—oxygen extraction index; IVO_2—oxygen consumption index; IDO_2—oxygen delivery index; Lac—lactate; Glu—glucose; rSO_2—regional cerebral saturation. Values are shown as median and (25 - 75 quartile). Wilcoxson test, *—$p < 0.05$ comparing to the baseline.

Disclaimer/Publisher's Note: The statements, opinions and data contained in all publications are solely those of the individual author(s) and contributor(s) and not of MDPI and/or the editor(s). MDPI and/or the editor(s) disclaim responsibility for any injury to people or property resulting from any ideas, methods, instructions or products referred to in the content.
